# Multilayer Carbon-Structured BaTiO_3_@C Nanocomposites with Wide Microwave Absorption Bandwidth and Excellent Corrosion Resistance

**DOI:** 10.3390/ma19102032

**Published:** 2026-05-13

**Authors:** Sichen Guo, Yijing Sun, Shanxin Li, Xuzhou Jiang, Dongbai Sun

**Affiliations:** 1 School of Materials Science and Engineering, Sun Yat-sen University & Southern Marine Science and Engineering Guangdong Laboratory (Zhuhai), Guangzhou 510006, China; 2 Sino-French Institute of Nuclear Engineering & Technology, Sun Yat-sen University, Zhuhai 519082, China

**Keywords:** nanocomposite, microwave absorption, corrosion resistance

## Abstract

Developing lightweight materials that simultaneously achieve efficient electromagnetic wave absorption and robust corrosion resistance remains a significant challenge for marine stealth and electromagnetic protection applications. The main obstacle lies in the rational integration of electromagnetic attenuation capability, impedance matching, and corrosion protection. In this work, a multilayer carbon-structured BaTiO_3_@C nanocomposite (CSTB-x) was successfully fabricated via freeze-drying combined with in situ pyrolysis. During the carbonization process, chitosan (CS) was transformed into a nitrogen-doped multilayer porous carbon framework, while BaTiO_3_ particles were embedded into the carbon matrix to construct a BaTiO_3_@C heterostructure. Benefiting from optimized impedance matching and the synergistic contributions of conduction loss, dipolar polarization, and interfacial polarization, CSTB-1.0 delivered a minimum reflection loss (RL_min_) of −48.07 dB at 6.16 GHz with a thickness of 3.32 mm, and achieved a maximum effective absorption bandwidth (EAB) of 7.04 GHz at a thickness of 1.88 mm. In addition, CSTB-1.0 exhibited a low corrosion current density (8.93 × 10^−6^ A/cm^2^) and a high polarization resistance (7.87 × 10^3^ Ω∙cm^2^), indicating excellent corrosion protection performance. The enhanced corrosion resistance is mainly attributed to the barrier effect of the multilayer carbon framework and the tortuous diffusion pathways generated by the porous and core–shell structures. Moreover, the material showed a minimum radar cross-section (RCS) value of −41.25 dBsm, demonstrating remarkable electromagnetic scattering suppression capability. These results provide a feasible strategy for the design and fabrication of marine stealth materials with integrated microwave absorption and corrosion resistance.

## 1. Introduction

With the rapid advancement of communication technologies such as 5G systems and radar detection, electromagnetic (EM) pollution and interference have become increasingly serious, posing potential risks to both electronic devices and human health [1,2]. This demand is particularly urgent in military ships, marine equipment, and offshore engineering facilities, where materials are expected not only to deliver effective radar stealth performance but also to operate reliably in harsh marine environments with high salinity, high humidity, and abundant corrosive ions [3]. Therefore, the development of multifunctional materials that simultaneously integrate electromagnetic wave (EMW) absorption and corrosion resistance has become an important yet challenging research topic.

However, the design of EMW absorbing materials for marine environments still faces several significant challenges. On the one hand, efficient EMW absorption relies on good impedance matching and the synergistic contribution of multiple loss mechanisms, including conduction loss, dipolar polarization, and interfacial polarization [4]. In practical systems, however, enhanced conductivity or increased dielectric response often leads to impedance mismatch, which restricts the penetration of incident electromagnetic waves into the absorber [5]. On the other hand, most existing studies have mainly focused on optimizing microwave absorption metrics such as reflection loss and effective absorption bandwidth, while insufficient attention has been paid to the corrosion protection capability of materials in salt spray or chloride-containing environments [6]. As a result, how to achieve the synergistic optimization of EMW absorption performance and environmental stability in lightweight material systems remains a key issue in current research.

Chitosan-derived carbon materials have attracted extensive attention in the field of EMW absorption because of their low density, renewability, abundant nitrogen-containing functional groups, and tunable porous structures [7,8]. After pyrolysis, the resulting nitrogen-doped carbon framework can provide continuous conductive pathways, abundant defect sites, and dipolar polarization centers, while the porous structure is beneficial for prolonging the propagation path of electromagnetic waves and promoting multiple scattering [8,9]. In addition, the carbonized chitosan-derived structure can also act as a physical barrier, thereby improving corrosion protection to some extent [10,11]. For example, a TiB_2_-chitosan coating has been reported to exhibit an I_corr_ value of 1.37 × 10^−5^ A/cm^2^ [11]. BaTiO_3_, as a typical perovskite ferroelectric ceramic, possesses a high dielectric constant, excellent polarization capability, and tunable physicochemical properties. Recently, BaTiO_3_-based materials have attracted increasing attention in multifunctional applications, and their properties can be optimized through dopant engineering, heterostructure construction, and crystal-structure regulation. For example, Rh doping has been used to improve the photocatalytic activity of BaTiO_3_, BaTiO_3_/CuO nanocomposites have been developed for enhanced photocatalytic performance, and highly tetragonal BaTiO_3_ nanoparticles have been obtained by regulating aging time and calcination temperature [12,13,14]. However, pristine BaTiO_3_ shows a reflection loss of only 21.8 dB and an absorption bandwidth of 1.7 GHz [15], indicating its limited attenuation capability [16,17]. Meanwhile, single-component chitosan-derived carbon usually suffers from impedance mismatch due to its relatively high conductivity. Therefore, combining BaTiO_3_ with chitosan-derived carbon is expected to enhance interfacial polarization, regulate dielectric response, and improve impedance matching through the construction of heterogeneous interfaces, thereby enabling the synergistic enhancement of microwave absorption and corrosion resistance [16,18,19].

To address the difficulty of simultaneously optimizing electromagnetic loss, impedance matching, and environmental stability in lightweight EMW absorbing materials, a multilayer carbon-structured BaTiO_3_@C nanocomposite was constructed in this work through a freeze-drying combined with in situ pyrolysis strategy. The chitosan-derived nitrogen-doped porous carbon framework provides abundant defect sites as well as barrier protection, while the in situ formed BaTiO_3_@C core–shell heterointerfaces introduce tunable polarization-active sites into the system. By adjusting the BaTiO_3_ content, the synergistic optimization of polarization loss, conduction loss, and impedance matching was achieved. As a result, the obtained material not only exhibits excellent microwave absorption performance, but also shows promising radar scattering suppression capability and corrosion protection potential. This work provides a new strategy for the design of lightweight multifunctional stealth and protective materials for marine environments.

## 2. Materials and Methods

### 2.1. Materials

Chitosan (CS) was purchased from Macklin Inc. (Shanghai, China). BaTiO_3_ (TB) and acetic acid were purchased from Aladdin Co., Ltd. (Shanghai, China). The 5 wt% Nafion solution was purchased from Du Pont China Holding Co., Ltd. (Shenzhen, China). The isopropanol was purchased from Guangzhou Chemical Reagent Factory (Guangzhou, China). Sodium chloride (NaCl) was purchased from General-Reagent Co., Ltd. (Shanghai, China). Paraffin wax was purchased from Shandong Xiya Chemical Co., Ltd. (Linyi, China). All reagents were used without further purification.

### 2.2. Fabrication of Multilayer Carbon-Structured BaTiO_3_@C Nanocomposites

First, x g (x = 0, 0.5, 1, and 1.5) of BaTiO_3_ was added to 99 mL of deionized water and thoroughly dispersed under stirring. Then, 3 g of chitosan was introduced and the mixture was continuously stirred to ensure homogeneous mixing. Next, 1 mL of acetic acid was added to promote the dissolution of chitosan and form a uniform colloidal solution. After continuous stirring for 4 h, stable colloids with different BaTiO_3_ contents were obtained. The resulting colloidal solutions were then freeze-dried for 24 h to obtain TB/CS aerogel precursors. Finally, pyrolysis was carried out starting from 20 °C, heating to 800 °C at a rate of 2 °C/min, holding at this temperature for 2 h, and then cooling to 20 °C at a rate of 2 °C/min. According to the different amounts of BaTiO_3_ added, the obtained multilayer carbon-structured BaTiO_3_@C nanocomposites were designated as CSTB-0, CSTB-0.5, CSTB-1.0, and CSTB-1.5, respectively.

### 2.3. Characterization

Characterization of the multilayer carbon-structured BaTiO_3_@C nanocomposites: The instruments and methods used for material characterization are described in the Supporting Information.

Electromagnetic parameter measurements: The multilayer carbon-structured BaTiO_3_@C nanocomposites (35 wt%) were mixed with paraffin wax and pressed into hollow cylindrical specimens (dout = 7.0 mm, din = 3.04 mm). The electromagnetic parameters were measured using a vector network analyzer (VNA, MS46522B, Anritsu Corporation, Atsugi, Japan) across the frequency range of 2.0–18.0 GHz. Since neither BaTiO_3_ nor chitosan-derived carbon contains any magnetic element (such as Fe, Co or Ni), the resulting CSTB-x composites are intrinsically non-magnetic, with relative complex permeability satisfying $\mu^{\prime }=1$, $\mu^{\prime \prime }=\;0$. Therefore, only the complex permittivity was measured, in line with the convention adopted in similar studies of BaTiO_3_–carbon-based dielectric absorber systems [19,20].

Corrosion resistance measurements: The electrochemical behavior of the electrodes was evaluated in 3.5 wt% NaCl solution using a three-electrode system (CorrTest CS100, Wuhan Corrtest Instruments Co., Ltd., Wuhan, China). The open-circuit potential (OCP) was recorded for 2400 s. Tafel measurements were carried out over the potential range from −0.5 V to 0.5 V, and the polarization curves were recorded at a scan rate of 1 mV/s. Electrochemical impedance spectroscopy (EIS) was performed over the frequency range from 100 kHz to 0.01 Hz. Detailed experimental information is provided in the Supporting Information.

The microwave absorption data were processed using MATLAB R2023a (The MathWorks, Inc., Natick, MA, USA). The monostatic RCS simulation was performed using FEKO software (Altair Engineering Inc., Troy, MI, USA). The EIS equivalent-circuit fitting was performed using ZSimpWin software (AMETEK Scientific Instruments, Berwyn, PA, USA). All figures were prepared using Origin 2026 (OriginLab Corporation, Northampton, MA, USA).

## 3. Results and Discussion

### 3.1. Structure and Morphology Characterization

**Figure 1:**
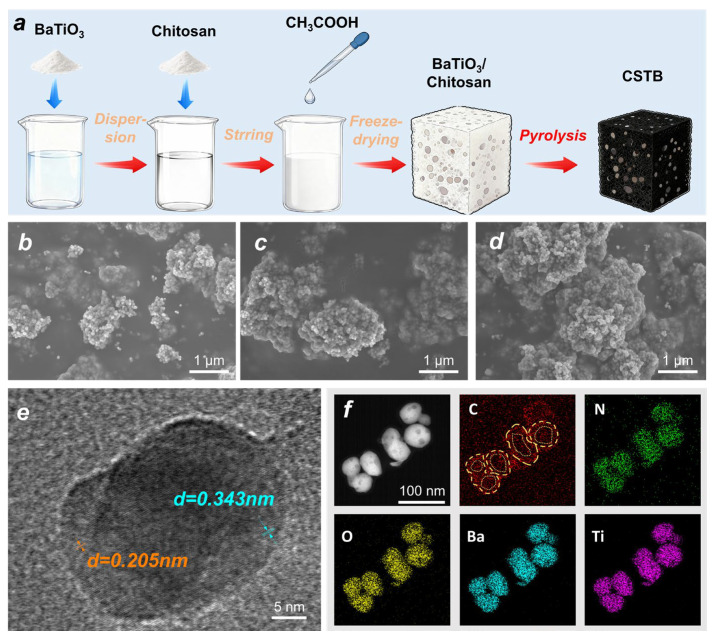
(**a**) Schematic diagram of the preparation of BaTiO_3_@C nanocomposites. SEM images of (**b**) CSTB-0.5, (**c**) CSTB-1.0 and (**d**) CSTB-1.5. (**e**) HRTEM images, and (**f**) Elemental composition of CSTB-1.0.

In this study, a multilayer carbon-structured BaTiO_3_@C nanocomposite was successfully synthesized via freeze-drying combined with in situ pyrolysis, and the fabrication process is illustrated in Figure 1a. During pyrolysis, chitosan gradually decomposed and carbonized, forming a multilayer carbon network structure. Meanwhile, BaTiO_3_ particles were dispersed on the surface of the carbon network, and a certain degree of aggregation appeared at higher loading levels. The SEM images of CSTB-0, CSTB-0.5, CSTB-1.0, and CSTB-1.5 shown in Figure S1 and Figure 1b–d reveal that, with increasing BaTiO_3_ content, the number of particles on the surface of the carbon framework gradually increased. As shown in Figure S2, the low-magnification SEM images reveal that BaTiO_3_ particles are generally distributed over the chitosan-derived carbon framework without obvious large-scale phase separation, indicating acceptable structural uniformity of the CSTB-x composites. At higher magnification, slight local aggregation can be observed, especially for samples with higher BaTiO_3_ loading. This phenomenon is mainly attributed to the high specific surface area and strong van der Waals interactions of nanoscale or submicron-scale BaTiO_3_ particles during drying and carbonization. Nevertheless, chitosan can act as a dispersing and supporting matrix through interactions between its amino/hydroxyl groups and BaTiO_3_ particle surfaces, which helps improve the dispersion of BaTiO_3_ and ensures good reproducibility of the composite structure [21]. At the same time, the HRTEM image and STEM-EDS elemental mapping results in Figure 1e–f further confirm the formation of the BaTiO_3_@C core–shell heterostructure in CSTB-1.0. Clear lattice fringes with interplanar spacings of 0.205 nm and 0.343 nm can be observed, which are attributed to BaTiO_3_ and graphitic carbon, respectively. More importantly, the elemental mapping results show that Ba, Ti, and O are mainly distributed in the inner region of the particles, whereas C is densely distributed around the outer region and relatively weak in the central region. This shell-like distribution of C provides direct evidence that the carbon layer is coated on the surface of BaTiO_3_ particles. Therefore, the re-collected elemental mapping results demonstrate that the BaTiO_3_@C core–shell structure is representative of the CSTB-1.0 sample rather than being only a local observation from a single TEM image. After carbonization at 800 °C under a nitrogen atmosphere, chitosan was transformed into a three-dimensional porous carbon framework, which provided pathways for electron transport, thus helping improve the electrical conductivity of the material and enhance conduction loss [18,22]. Meanwhile, as a typical dielectric polarization unit, BaTiO_3_ particles could induce interfacial polarization and dipolar polarization under an alternating electromagnetic field, thereby further strengthening polarization loss and promoting microwave energy dissipation [18,19,23].

### 3.2. Pore Structure, Phase Composition and Surface Chemical States

**Figure 2:**
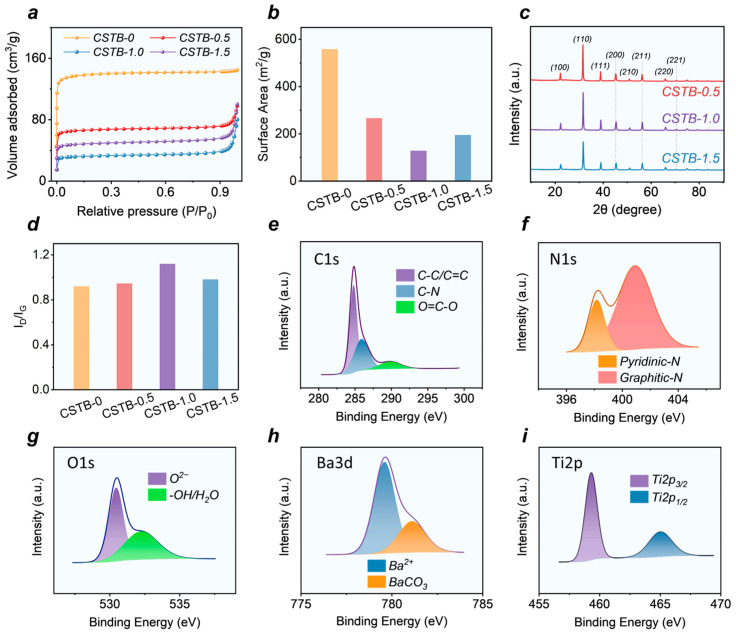
(**a**) N_2_ adsorption/desorption isotherms, (**b**) BET surface area. (**c**) XRD patterns. (**d**) ID/IG of the BaTiO_3_@C nanocomposites. XPS spectra of (**e**) C1s, (**f**) N1s, and (**g**) O1s, (**h**) Ba3d, (**i**) Ti2p.

All samples exhibited type IV nitrogen adsorption–desorption isotherms with distinct hysteresis loops, indicating that the as-prepared multilayer carbon-structured BaTiO_3_@C nanocomposites are predominantly mesoporous (Figure 2a) [24,25]. As shown in Figure 2b, with increasing BaTiO_3_ content, the specific surface area did not change monotonically, but instead first decreased significantly, reaching a minimum value of 127.9 m^2^/g for CSTB-1.0, and then increased again in CSTB-1.5. This variation may be related to two factors. On the one hand, BaTiO_3_, which has a relatively low specific surface area, exerts a dilution effect on the high-specific-surface-area carbon component. On the other hand, the introduction of BaTiO_3_ affects the pore-forming process of the carbon framework, resulting in the loss of part of the pore structure or an increase in pore size [26]. Such structural evolution may reduce the charge transport pathways in the material, thereby tuning the dielectric constant of the sample to a more reasonable range [17,27]. When the BaTiO_3_ content is further increased, carbon may attach around the BaTiO_3_ particles in the form of thin layers or shells, thereby increasing the specific surface area of the sample to some extent [28]. It should be noted that there is no simple positive correlation between microwave absorption performance and specific surface area [29]. In contrast, moderate pore structure regulation, a rational dielectric response, and enhanced interfacial polarization are more favorable for achieving excellent impedance matching and broadband absorption. Therefore, although CSTB-1.0 has the lowest specific surface area, its enlarged average pore size and moderate averaged dielectric parameters still contribute to improved impedance matching. As summarized in Table S1, CSTB-1.0 exhibits the largest average pore size of 3.8730 nm, with a specific surface area of 127.9394 m^2^/g and a pore volume of 0.1239 cm^3^/g. This indicates that the appropriate introduction of BaTiO_3_ regulates the highly porous carbon framework and avoids an excessively conductive network, while still maintaining sufficient porous channels and polarization-active interfaces.

The XRD results are shown in Figure 2c. Compared with the pure chitosan-derived carbon sample (CSTB-0, Figure S4), the composite also exhibits a diffraction peak at 45.4°, corresponding to the (200) crystal plane of BaTiO_3_ [30], indicating that the composite is composed of both barium titanate and carbon phases. Raman spectra (Figure S5) further reveal the defect characteristics of the carbon component. It is well known that the D band and G band correspond to structural defects in the graphite lattice and ordered sp2 carbon structures, respectively [31]. As shown in Figure 2d, the ID/IG value of the samples first increased and then decreased with increasing BaTiO_3_ content, reaching a maximum of 1.12 for CSTB-1.0 and then dropping to 0.98 for CSTB-1.5. These results indicate that the introduction of an appropriate amount of BaTiO_3_ is beneficial for generating more defect sites, whereas excessive BaTiO_3_ may lead to particle aggregation and interfacial saturation, causing the carbon layer to gradually evolve into a more continuous coating structure accompanied by a certain degree of structural rearrangement, thereby reducing the defect density and increasing the local graphitization degree [32,33].

Figure 2e–i present the XPS results of the samples. The C1s spectrum shows three characteristic peaks corresponding to C-C/C=C, C-N, and O=C-O, indicating the presence of multiple chemical bonding states in the carbon framework [34,35,36]. The N1s spectrum reveals the formation of pyridinic-N and graphitic-N, confirming the successful doping of nitrogen into the carbon framework. These nitrogen-containing sites help introduce dipolar polarization centers and enhance dielectric loss [37]. The O1s spectrum shows peaks originating from lattice oxygen O^2−^ and surface-adsorbed oxygen species (-OH/H_2_O) [8,38]. The Ba3d spectrum displays two types of Ba^2+^ associated with the BaTiO_3_ lattice and surface barium carbonate [34,38,39], while the Ti2p spectrum shows that the Ti species in the sample correspond to the Ti2p3/2 and Ti2p1/2 chemical states of Ti^4+^ [25].

### 3.3. Microwave Absorption Performance

To evaluate the microwave absorption performance of the multilayer carbonstructured BaTiO_3_@C nanocomposites, the reflection loss (RL) and effective absorption bandwidth (EAB) of all samples were calculated based on transmission line theory [39]. The input impedance $Z_{in}$ and RL can be expressed as follows:

(1) $$Z_{in}=Z_{0}\left(\sqrt{\frac{\mu_{r}}{\varepsilon_{r}}}\right)tanh\left[j\left(\frac{2\pi fd}{c}\right)\left(\sqrt{\mu_{r}\varepsilon_{r}}\right)\right]$$

(2) $$RL=20{log}_{10}\left|\frac{Z_{in}-Z_{0}}{Z_{in}+Z_{0}}\right|$$

where $Z_{in}$ and $Z_{0}$ denote the input impedance and spatial impedance, $\varepsilon_{r}$ $\left(\varepsilon_{r}=\varepsilon^{\prime }-j\varepsilon^{\prime \prime }\right)$ and $\mu_{r}$ ($\mu_{r}$ = $\mu^{\prime }$−$j\mu^{\prime \prime }$) represent complex permittivity and complex permeability, c, d, f, and j denote the speed of light, the thickness of the absorber, the frequency, and the imaginary units, respectively. Since the CSTB-x composites are non-magnetic dielectric absorbers, their microwave absorption behavior is mainly governed by the complex permittivity. In this work, the EAB is defined as the frequency range where RL < −10 dB, corresponding to more than 90% attenuation of incident electromagnetic wave energy.

**Figure 3:**
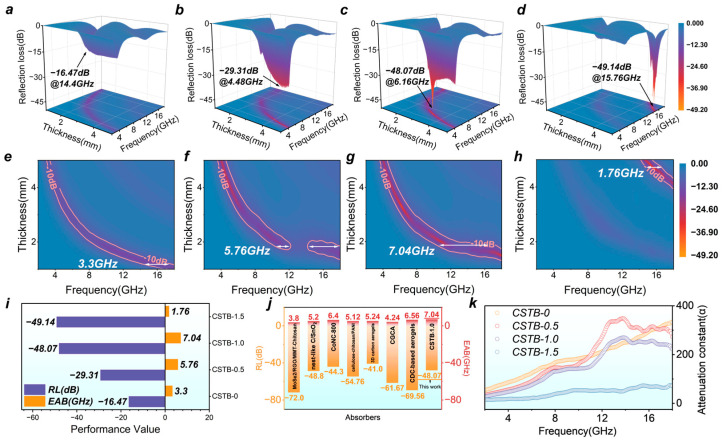
RL of (**a**) CSTB-0, (**b**) CSTB-0.5, (**c**) CSTB-1.0, and (**d**) CSTB-1.5. EAB of (**e**) CSTB-0, (**f**) CSTB-0.5, (**g**) CSTB-1.0 and (**h**) CSTB-1.5. (**i**) Summary histogram of RL_min_ and EAB_max_, (**j**) Comparison of microwave absorption performance of similar absorbers, and (**k**) Attenuation constant (α) of the BaTiO_3_@C nanocomposites.

Figure 3a–d present the three-dimensional RL distributions of the different samples. CSTB-0 achieved an RL_min_ of −16.47 dB at 14.4 GHz with a thickness of 1.3 mm, while CSTB-0.5 reached −29.31 dB at 4.48 GHz with a thickness of 4.52 mm. CSTB-1.0 exhibited an RL_min_ of −48.07 dB at 6.16 GHz with a thickness of 3.32 mm. CSTB-1.5 reached −49.14 dB at 15.76 GHz with a thickness of 4.67 mm. Although CSTB-1.5 showed an absorption peak comparable to that of CSTB-1.0, its operating frequency was shifted to a higher band and required a greater matching thickness.

Figure 3e–h illustrate the two-dimensional color maps of EAB. The EAB_max_ values of CSTB-0, CSTB-0.5, and CSTB-1.5 were 3.3 GHz, 5.76 GHz, and 1.76 GHz, respectively, with corresponding matching thicknesses of 1.19 mm, 1.84 mm, and 4.73 mm. Notably, CSTB-1.0 achieved an EAB_max_ of 7.04 GHz at a low thickness of 1.88 mm, demonstrating superior broadband absorption capability. Figure 3i further summarizes the microwave absorption performance of all samples, clearly showing that CSTB-1.0 exhibited the best overall performance. In particular, compared with several representative chitosan-derived microwave-absorbing materials (Figure 3j, Table S2), the multilayer carbon-structured BaTiO_3_@C nanocomposite with an appropriate BaTiO_3_ content developed in this work shows a clear advantage in effective absorption bandwidth.

The attenuation constant α was further calculated to characterize the intrinsic attenuation capability of the absorber toward incident microwaves:

(3) $$\alpha =\frac{\sqrt{2}\pi f}{c}\sqrt{\left(\mu^{\prime \prime }\varepsilon^{\prime \prime }-\mu^{\prime }\varepsilon^{\prime }\right)+\sqrt{{\left(\mu^{\prime \prime }\varepsilon^{\prime \prime }-\mu^{\prime }\varepsilon^{\prime }\right)}^{2}+{\left(\mu^{\prime }\varepsilon^{\prime \prime }+\mu^{\prime \prime }\varepsilon^{\prime }\right)}^{2}}}$$

where $\varepsilon^{\prime }$ and $\varepsilon^{\prime \prime }$ are the real and imaginary parts of the complex permittivity, while $\mu^{\prime }$ and $\mu^{\prime \prime }$ are the real and imaginary parts of the complex permeability, respectively. A larger α value generally indicates stronger electromagnetic energy attenuation inside the absorber. As shown in Figure 3k, compared with the pure chitosan-derived carbon sample CSTB-0, the BaTiO_3_@C/carbon heterostructure formed after the introduction of BaTiO_3_ significantly enhanced the electromagnetic attenuation capability of the material. However, it should be emphasized that a higher attenuation constant does not necessarily correspond to the best microwave absorption performance. Only when attenuation capability and impedance matching are synergistically optimized can the material achieve superior overall microwave absorption performance.

### 3.4. Electromagnetic Loss Mechanism

To further clarify the microwave absorption mechanism of the CSTB-x system, its impedance matching and dielectric loss behaviors were analyzed. Impedance matching was evaluated using the impedance matching index (IMI), which describes the deviation between the input impedance of the absorber and the impedance of free space [39,40,41,42,43,44]:

(4) $$IMI=\left|\frac{Z_{in}-Z_{0}}{Z_{0}}\right|$$

**Figure 4:**
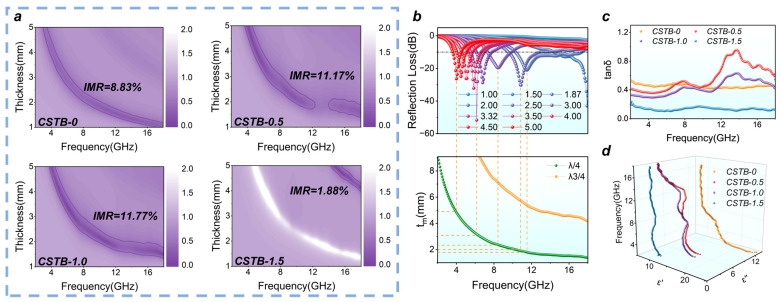
(**a**) Impedance matching indicator, (**b**) λ/4 model of CSTB-1.0, (**c**) dielectric loss tangent of all samples and (**d**) Cole-Cole semicircles of all samples.

A smaller IMI value indicates a lower impedance mismatch between the absorber and free space, which is favorable for the entry of incident electromagnetic waves into the absorber. In this work, the effective impedance matching region was identified using IMI < 0.5. As shown in Figure 4a, the effective impedance matching region of CSTB-1.0 is larger than that of the other samples, indicating that it can achieve efficient electromagnetic wave incidence over a broader frequency range.

To quantitatively compare the impedance matching behavior, the impedance matching region (IMR) was defined as the percentage of calculated frequency–thickness points satisfying IMI < 0.5. As summarized in Table S1, CSTB-1.0 exhibits the highest IMR value of 11.77%, higher than CSTB-0 (8.83%), CSTB-0.5 (11.17%), and CSTB-1.5 (1.88%). The IMR of CSTB-1.0 is approximately 6.26 times that of CSTB-1.5, demonstrating that CSTB-1.0 possesses a broader effective impedance matching region.

In addition, the relationship between the matching thickness and matching frequency was analyzed using the quarter-wavelength (λ/4) cancellation model:

(5) $$t_{m}=\frac{n\lambda }{4}=\frac{nc}{4f_{m}\sqrt{\left|\mu_{r}\right|\left|\varepsilon_{r}\right|}}\;(n=1,3,5,\ldots )$$

where $t_{m}$ denotes the matching thickness of the absorber corresponding to the reflection loss minimum, n is an odd integer, c is the speed of light in vacuum, $f_{m}$ represents the matching frequency, and $\left|\mu_{r}\right|\left|\varepsilon_{r}\right|$ is the product of the absolute values of the complex relative permeability and complex relative permittivity. When the absorber thickness satisfies this condition, the reflected waves from the absorber surface and the metal backing can cancel each other, leading to enhanced microwave attenuation. As shown in Figure 4b, the experimental matching thickness agrees well with the λ/4 model, suggesting that the microwave absorption behavior of this system follows the interference cancellation mechanism. By reducing surface reflection and enhancing the propagation of electromagnetic waves within the material, this mechanism further improves the absorption efficiency [45].

The dielectric loss tangent tanδ was used to evaluate the dielectric loss capability of the CSTB-x samples:

(6) $$tan\delta =\frac{\varepsilon^{\prime \prime }}{\varepsilon^{\prime }}$$

where tanδ is the dielectric loss tangent. $\varepsilon^{\prime }$ is the real part of the dielectric constant, and $\varepsilon^{\prime \prime }$ is the imaginary part of the dielectric constant. δ is the loss angle. Therefore, a higher tanδ generally indicates stronger dielectric loss. As shown in Figure 4c, the tanδ values of CSTB-0.5 and CSTB-1.0 are higher than those of the other samples, and the relatively high tanδ peaks observed in the high-frequency region originate from the interfacial polarization relaxation induced by the BaTiO_3_@C core–shell heterointerface [46,47]. Notably, although CSTB-1.0 does not exhibit the highest attenuation constant α (Figure 3k) or dielectric loss tangent tanδ (Figure 4c), it still achieves the best microwave absorption performance owing to its superior impedance matching. This indicates that, for this system, excellent microwave absorption performance arises from the synergistic balance between loss capability and impedance matching, rather than from maximizing a single loss parameter [19,48].

To further support this conclusion quantitatively, the pore-structure and electromagnetic parameters of the CSTB-x samples are summarized in Table S1. The dielectric parameters and attenuation constant listed in Table S1 are average values over the measured frequency range of 2–18 GHz. CSTB-0 shows relatively high $\bar{\varepsilon^{\prime }}$ and $\bar{\varepsilon^{\prime \prime }}$ values of 17.7682 and 8.1177, respectively, which may result in excessive dielectric loss and strong surface reflection. In contrast, CSTB-1.5 exhibits much lower averaged dielectric parameters, with $\bar{\varepsilon^{\prime }}$ and $\bar{\varepsilon^{\prime \prime }}$ values of 9.5732 and 1.3133, respectively, and a low $\bar{\tan\delta_{\varepsilon }}$ value of 0.1368, suggesting insufficient electromagnetic attenuation. CSTB-1.0 presents moderate $\bar{\varepsilon^{\prime }}$ and $\bar{\varepsilon^{\prime \prime }}$ values of 11.8968 and 4.9423, together with a moderate $\bar{\tan\delta_{\varepsilon }}$ value of 0.4325. Meanwhile, its average attenuation constant $\bar{\alpha }$ is 155.0398, indicating sufficient attenuation capability while avoiding excessive loss-induced impedance mismatch. Therefore, CSTB-1.0 achieves a more suitable balance between dielectric loss, attenuation capability, and electromagnetic wave penetration.

According to Debye relaxation theory, the relationship between $\varepsilon^{\prime }$ and $\varepsilon^{\prime \prime }$ can be described by the Cole–Cole semicircle model:

(7) $${\left(\varepsilon^{\prime }-\frac{\varepsilon_{s}+\varepsilon_{\infty }}{2}\right)}^{2}+{\left(\varepsilon^{\prime \prime }-\frac{\sigma }{{\omega \varepsilon }_{0}}\right)}^{2}={\left(\frac{\varepsilon_{s}-\varepsilon_{\infty }}{2}\right)}^{2}$$

where $\varepsilon_{s}$ is the static permittivity, $\varepsilon_{\infty }$ is the relative permittivity at the high-frequency limit, σ is the electrical conductivity, ω is the angular frequency, and $\varepsilon_{0}$ is the vacuum permittivity. In this model, each semicircle generally corresponds to one Debye-type polarization relaxation process, while deviations from ideal semicircles are related to conductive loss and multiple relaxation behaviors. Therefore, Cole–Cole plots can be used to analyze the polarization relaxation behavior of microwave-absorbing materials [49].

As shown in Figure 4d, both CSTB-0.5 and CSTB-1.0 exhibit obvious multiple polarization relaxation features, indicating the presence of abundant interfacial polarization and dipolar polarization processes within the materials [18]. These polarization processes mainly arise from the combined effects of the BaTiO_3_@C heterointerfaces and internal defects such as nitrogen doping [50,51,52]. Among them, CSTB-1.0, benefiting from a more rational pore structure, more abundant defect-induced polarization centers, and a moderately tuned dielectric response (Figure S3 and Table S1), achieves a better balance between polarization loss and impedance matching, and therefore exhibits superior electromagnetic attenuation capability and broadband absorption performance [17,27].

The optimal microwave absorption performance of CSTB-1.0 can therefore be understood from the quantitative balance between electromagnetic wave penetration and energy attenuation. For CSTB-0, the continuous chitosan-derived carbon framework provides strong conduction loss, but the high $\bar{\varepsilon^{\prime }}$, $\bar{\varepsilon^{\prime \prime }}$ and $\bar{\alpha }$ values may induce severe impedance mismatch and surface reflection, which limits the effective incidence of electromagnetic waves into the absorber. With the introduction of BaTiO_3_, the conductive network is regulated and abundant BaTiO_3_@C heterointerfaces are formed, providing additional interfacial polarization sites and improving dielectric relaxation. However, excessive BaTiO_3_ loading in CSTB-1.5 weakens dielectric loss and attenuation capability, as reflected by its low $\bar{\varepsilon^{\prime \prime }}$, low $\bar{\tan\delta_{\varepsilon }}$, low $\bar{\alpha }$, and small IMR. In contrast, CSTB-1.0 simultaneously exhibits moderate averaged dielectric parameters, sufficient averaged attenuation capability, and the highest IMR value, allowing more incident electromagnetic waves to enter the absorber and be efficiently dissipated through conduction loss, dipolar polarization, interfacial polarization, and multiple scattering within the porous multilayer structure. As a result, CSTB-1.0 achieves the best overall microwave absorption performance among all samples.

**Figure 5:**
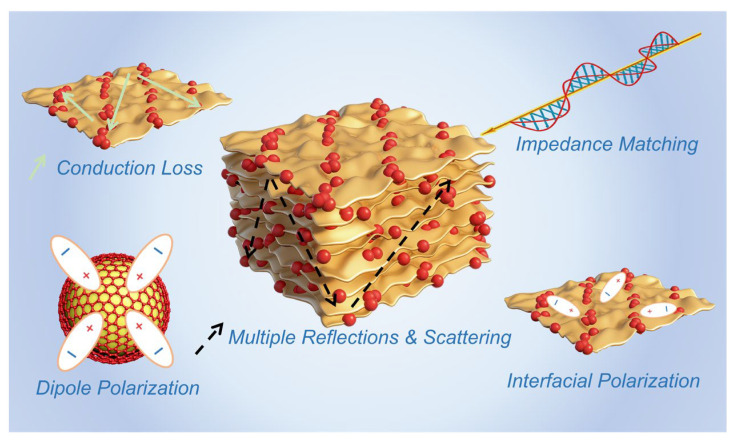
The microwave absorption mechanism of the multilayer carbon-structured BaTiO_3_@C nanocomposites.

Based on the above results, the microwave absorption mechanism of the multilayer carbon-structured BaTiO_3_@C nanocomposite can be summarized in the following three aspects (Figure 5):

(1) Optimized impedance matching. The introduction of an appropriate amount of BaTiO_3_ effectively tunes the complex permittivity of the system, thereby avoiding the severe mismatch caused by the excessively high dielectric constant of the pure carbon system and allowing more incident electromagnetic waves to enter the interior of the material. This is the prerequisite for CSTB-1.0 to achieve broadband absorption and efficient attenuation.

(2) Enhanced polarization loss. The BaTiO_3_@C core–shell heterointerfaces provide a large number of interfacial polarization sites, while the defects and nitrogen doping within the multilayer carbon framework introduce abundant dipolar polarization centers. Charges accumulate and relax at the heterointerfaces and internal defect sites, thereby effectively dissipating electromagnetic energy.

(3) Moderate conduction loss. The chitosan-derived multilayer carbon network provides continuous pathways for electron migration, which is beneficial for enhancing conduction loss. Meanwhile, as shown in Figure S3, regulating the BaTiO_3_ content can effectively reduce the imaginary part of the complex permittivity, thereby tuning the conductive network and dielectric response. This allows the material to maintain moderate conductivity and thus realize the synergistic optimization of conduction loss and impedance matching [52].

In summary, by regulating the BaTiO_3_ content, the synergistic optimization of impedance matching, polarization loss, and conduction loss was achieved in the nanocomposite, enabling CSTB-1.0 to exhibit the best overall microwave absorption performance.

### 3.5. Application-Oriented Evaluation: RCS Simulation and Corrosion Resistance

**Figure 6:**
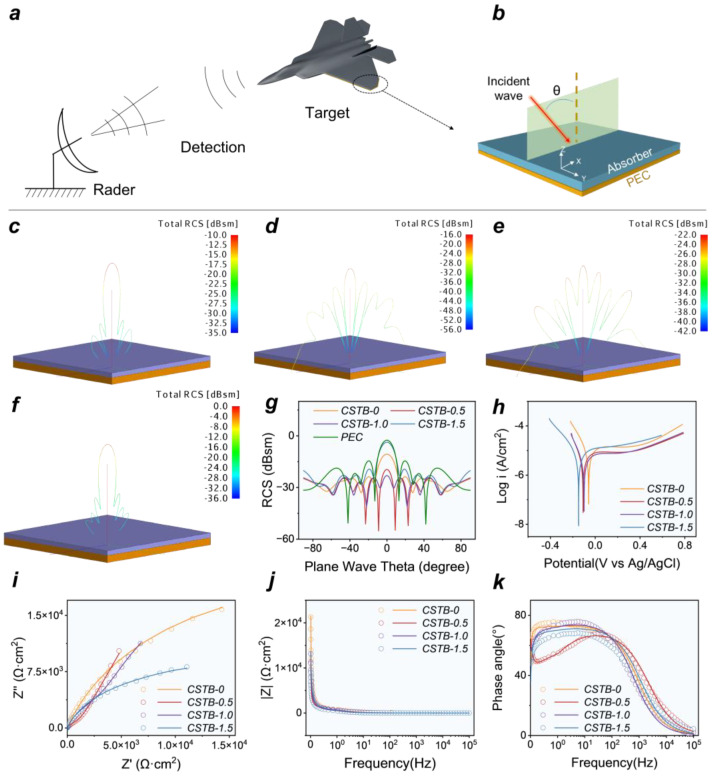
(**a**) Aircraft monostatic scattering RCS, (**b**) RCS simulation model, corresponding RCS simulation results of (**c**) CSTB-0, (**d**) CSTB-0.5, (**e**) CSTB-1.0, (**f**) CSTB-1.5 and (**g**) RCS simulation curve comparison. (**h**) Tafel curves. EIS spectra and equivalent circuit fitting results: (**i**) Nyquist plots, (**j**) Bode modulus plots, and (**k**) phase-angle plots in 3.5 wt% NaCl solution. The symbols and solid lines represent the experimental and fitted data, respectively.

To evaluate the effectiveness of the multilayer carbon-structured BaTiO_3_@C nanocomposites in radar stealth applications, their monostatic radar cross section (RCS) was simulated using FEKO software (Altair Engineering Inc., Troy, MI, USA) (Figure 6a). RCS is a physical parameter used to characterize the echo intensity generated by a target under radar wave irradiation, and the RCS analysis of targets coated with microwave-absorbing materials is particularly important for stealth technology research. According to the metal-backed model [53], a simulation model consisting of an upper microwave-absorbing coating layer (CSTB-x, 3.32 mm) and a lower perfect electric conductor layer (PEC, 5.0 mm) was established (Figure 6b), with the plate size defined as 100 × 100 mm^2^. The operating frequency was set at 6.16 GHz, and the incident electromagnetic wave direction (−90° < θ < 90°) was located in the XOZ plane.

The simulation results are shown in Figure 6c–f, with a single PEC plate (Figure S6) selected as the control group. The results indicate that all CSTB-x absorbing coatings can reduce the RCS value of the target to different extents. When the incident angle θ is 0°, the RCS values decrease in the following order: PEC (−2.56 dBsm) > CSTB-1.5 (−3.70 dBsm) > CSTB-0 (−10.62 dBsm) > CSTB-0.5 (−19.57 dBsm) > CSTB-1.0 (−23.10 dBsm), indicating that CSTB-1.0 can effectively suppress the strong radar echoes from aircraft wall panels, such as wings and vertical tails, in the normal direction [54]. Notably, CSTB-1.0 exhibits relatively low RCS values over the incident angle range of −50° < θ < 50°, with a minimum value of −41.25 dBsm, and remains below −23 dBsm at all incident angles, demonstrating that this material not only possesses excellent scattering suppression capability but also maintains good stability under different incident angle conditions.

In addition, considering its potential marine application scenarios, the corrosion behavior of the samples in simulated seawater (3.5 wt% NaCl solution) was further investigated. A three-electrode electrochemical testing technique was employed, using the nanocomposite as the working electrode, and its corrosion resistance was evaluated by Tafel polarization curves (Figure 6h). The results show that CSTB-1.0 exhibits a relatively low I_corr_ value (8.93 × 10^−6^ A/cm^2^) and a relatively high polarization resistance (R_p_) value (7.87 × 10^3^ Ω∙cm^2^), indicating superior corrosion inhibition capability. Compared with the previously reported TiB2-chitosan coating (I_corr_ = 1.37 × 10^−5^ A/cm^2^) [11], CSTB-1.0 demonstrates better corrosion resistance in simulated seawater.

Figure 6i–k show the electrochemical impedance spectroscopy (EIS) spectra and corresponding equivalent circuit fitting results of the CSTB-x samples in 3.5 wt% NaCl solution. The hollow symbols represent the experimental data, while the solid lines represent the fitted results. To quantitatively analyze the corrosion-related electrochemical processes, equivalent circuit fitting was further performed using ZSimpWin software (AMETEK Scientific Instruments, Berwyn, PA, USA). Considering the different electrochemical responses of CSTB-0 and BaTiO_3_-containing CSTB composites, two equivalent circuits were used, as shown in Figure S7. For CSTB-0, the EIS spectrum was fitted using the one-time-constant circuit:

$$R_{s}+\left({CPE}_{c}∥R_{c}\right)$$

For CSTB-0.5, CSTB-1.0, and CSTB-1.5, the spectra were fitted using a two-time-constant equivalent circuit:

$$R_{s}+\left[{CPE}_{c}∥\left(R_{c}+\left({CPE}_{dl}∥R_{ct}\right)\right)\right]$$

In these circuits, $R_{s}$ represents the solution resistance, ${CPE}_{c}$ and $R_{c}$ are associated with the non-ideal capacitive response and resistance of the porous composite layer, and ${CPE}_{dl}$ and $R_{ct}$ correspond to the double-layer response and charge-transfer resistance at the electrolyte/electrode interface. The use of CPEs instead of ideal capacitors is appropriate for the porous and heterogeneous CSTB-x electrodes, where surface roughness, pore structure, and heterogeneous interfaces can lead to non-ideal capacitive behavior.

The fitted EIS parameters are summarized in Table S3. For CSTB-1.0, $R_{c}$ and $R_{ct}$ are 1.061 × 10^4^ $\Omega \cdot {cm}^{2}$ and 5.572 × 10^4^ $\Omega \cdot {cm}^{2}$, respectively. The total resistance contribution, estimated as $R_{c}+R_{ct}$, reaches 6.633 × 10^4^ $\Omega \cdot {cm}^{2}$, which is comparable to that of CSTB-0.5 and much higher than that of CSTB-1.5. In addition, CSTB-1.0 exhibits an impedance modulus of 3.03 × 10^4^ $\Omega \cdot {cm}^{2}$ at 0.01 Hz in the Bode plots, which is of the same order of magnitude as, and even higher than, that of the reported TiB_2_-chitosan coating, approximately 10^4^ $\Omega \cdot {cm}^{2}$ [11]. This comparison further indicates that the corrosion reaction process of CSTB-1.0 is effectively suppressed in 3.5 wt% NaCl solution. These results suggest that an appropriate BaTiO_3_ content can enhance the barrier effect of the multilayer carbon framework and suppress the interfacial charge-transfer process. In contrast, excessive BaTiO_3_ loading in CSTB-1.5 leads to a much lower $R_{ct}$ value, probably due to particle aggregation and the formation of more defective ion-diffusion pathways.

The Nyquist plots show broadly comparable capacitive arc radii, indicating that the apparent impedance responses of the CSTB-x samples are not dramatically different when judged only from the Nyquist plots [55]. Therefore, the corrosion resistance of CSTB-1.0 should not be evaluated solely based on the Nyquist arc radius. Instead, a comprehensive analysis combining Tafel polarization, Bode impedance modulus, phase-angle response, literature comparison, and equivalent circuit fitting is more appropriate. CSTB-1.0 exhibits the lowest corrosion current density, a relatively high polarization resistance, a high low-frequency impedance modulus comparable to reported chitosan-based anticorrosive coatings, and the highest total fitted resistance contribution among the CSTB-x samples. Moreover, CSTB-1.0 exhibits a larger phase angle, suggesting more pronounced capacitive behavior and a more effective barrier response against electrolyte penetration [56]. These results collectively demonstrate that CSTB-1.0 possesses the optimized corrosion protection performance.

The enhanced corrosion resistance of CSTB-1.0 is mainly attributed to the optimized porous/core–shell structure, in which the multilayer carbon framework acts as an effective physical barrier, while the BaTiO_3_@C heterostructure helps construct tortuous diffusion pathways. This structure delays the penetration of corrosive species and reduces the exposure of active interfaces to the electrolyte.

## 4. Conclusions

This study successfully constructed a multilayer carbon-structured BaTiO_3_@C nanocomposite through a combined freeze-drying and in situ pyrolysis strategy, and systematically investigated the effects of BaTiO_3_ content on its structural evolution, microwave absorption performance, and corrosion protection capability. The results demonstrate that the introduction of an appropriate amount of BaTiO_3_ can synergistically regulate interfacial polarization, dipolar polarization, conduction loss, and impedance matching. Benefiting from these synergistic effects, CSTB-1.0 achieves the best overall performance, exhibiting excellent microwave absorption capability, stable radar scattering suppression, and enhanced corrosion resistance. The improved performance is mainly attributed to the continuous conductive network and physical barrier effect provided by the multilayer carbon framework, as well as the enhanced polarization loss and optimized dielectric response induced by the BaTiO_3_@C heterointerfaces. This study indicates that the multilayer carbon-structured BaTiO_3_@C nanocomposite holds considerable promise for applications in microwave absorption and corrosion protection, and also provides a new strategy for the design of multifunctional electromagnetic protective materials for marine environments.

## Data Availability

The original contributions presented in this study are included in the article/Supplementary Material. Further inquiries can be directed to the corresponding authors.

## Supplementary Materials

The following supporting information can be downloaded at: https://www.mdpi.com/article/10.3390/ma19102032/s1, Figure S1. SEM image of CSTB-0; Figure S2. SEM images of the (a) CSTB-0.5, (b) CSTB-1.0 and (c) CSTB-1.5; Figure S3. The (a) real and (b) imaginary parts of the complex permittivity of the BaTiO_3_@C nanocomposites; Figure S4. XRD patterns of CSTB-0; Figure S5. Raman spectroscopy of the BaTiO_3_@C nanocomposites; Figure S6. Corresponding RCS simulation results of PEC; Figure S7. Equivalent circuits used for fitting the EIS spectra: (a) $R_{s}+\left({CPE}_{c}∥R_{c}\right)$ for CSTB-0 and (b) $R_{s}+\left[{CPE}_{c}∥\left(R_{c}+\left({CPE}_{dl}∥R_{ct}\right)\right)\right]$ for CSTB-0.5, CSTB-1.0, and CSTB-1.5; Table S1. Quantitative comparison of pore structure, average dielectric properties, average attenuation capability, and impedance matching behavior of BaTiO3@C nanocomposites; Table S2. Comparison of properties of some typical Chitosan-derived nanocomposites and similar metal/carbon-based absorbers; Table S3. EIS fitting parameters of CSTB-x samples in 3.5 wt% NaCl solution; Table S4. Fitted electrochemical parameters obtained from Tafel polarization curves of CSTB-x samples in 3.5 wt% NaCl solution. References [57,58,59,60,61,62,63,64] are cited in the Supplementary Materials.
