# Supplementary material for: Multilayer Carbon-Structured BaTiO3@C Nanocomposites with Wide Microwave Absorption Bandwidth and Excellent Corrosion Resistance
Source: Materials (Basel). 2026 May 13;19(10):2032. doi: 10.3390/ma19102032 (PMC13208703; doi:10.3390/ma19102032)
Supplement: Supplementary file 1 [file materials-19-02032-s001.zip › materials-4253521-supplementary.pdf]

Supplementary Material

Multilayer carbon-structured $BaTiO_3$@C nanocomposites with wide microwave absorption bandwidth and excellent corrosion resistance

Sichen Guoa, Yijing Sunb, *, Shanxin Lia, *, Xuzhou Jianga and Dongbai Suna, *

a School of Materials Science and Engineering, Sun Yat-sen University & Southern Marine Science and Engineering Guangdong Laboratory (Zhuhai), Guangzhou 510006, PR China
b Sino-French Institute of Nuclear Engineering & Technology, Sun Yat-Sen University, Zhuhai 519082, PR China
* Correspondence: sunyij5@mail.sysu.edu.cn (Y.S.); lishx86@mail.sysu.edu.cn (S.L.); sundongbai@mail.sysu.edu.cn (D.S.)

Supplementary Characterization

The microscopic morphology was characterized by scanning electron microscopy (SEM, Gemini 500) and the elemental composition was shown by an energy dispersive spectrometer (EDS, Oxford Instruments). The microstructure and the lattice striation information were provided by transmission electron microscopy (TEM, JEOL JEM2100). The phase composition was observed by X-ray diffractometer (XRD, D-MAX 2200 VPC). The state of carbon was recorded by Raman spectroscopy (Raman, inVia Qontor). The chemical state of the samples was measured by X-ray photoelectron spectroscopy (XPS, Thermo Fisher/ESCALAB Qxi). N2 adsorption–desorption isotherms and BET specific surface area were measured using a specific surface area and micropore analyzer (Micromeritics ASAP 2460).

Anti-corrosion Performance Measurements: The working electrode for corrosion measurements was prepared by drop-casting a mixed suspension of CSTB nanocomposites onto a glassy carbon electrode (3 mm diameter). Specifically, the suspension consisted of 2 mg CSTB nanocomposites, 10 μL 5 wt% Nafion solution, 100 μL isopropanol, and 300 μL deionized water. Thus, the CSTB nanocomposite was used in the form of a coating on the glassy carbon substrate, and Nafion served as the binder to improve the adhesion and stability of the coating. The exposed electrode area was 0.0707 cm^2, corresponding to the circular electrode surface with a diameter of 3 mm. Electrochemical behavior was determined in 3.5 wt% NaCl solution using a conventional three-electrode system connected to a CorrTest CS100 electrochemical workstation. Open-circuit potential (OCP) was monitored for 2400 s to ensure that the electrochemical system reached a stable state before further testing. Tafel polarization measurements were performed within a potential range from -0.5 to 0.5 V at a scan rate of 1 mV/s. Electrochemical impedance spectroscopy (EIS) measurements were conducted over a frequency range from 100 kHz to 0.01 Hz.

Supplementary Equations:

Debye-Scherrer equation [1,2]:

$$D = \frac{K\gamma}{B\ cos\theta} \qquad \text{(Eq. S1)}$$

where K is the Scherrer constant, D is the average thickness of the grain perpendicular to the crystal plane, B is the half-peak height width or integral width of the measured sample diffraction peak, θ is the Bragg angle, and γ is the X-ray wavelength, which is 1.54056 Å.

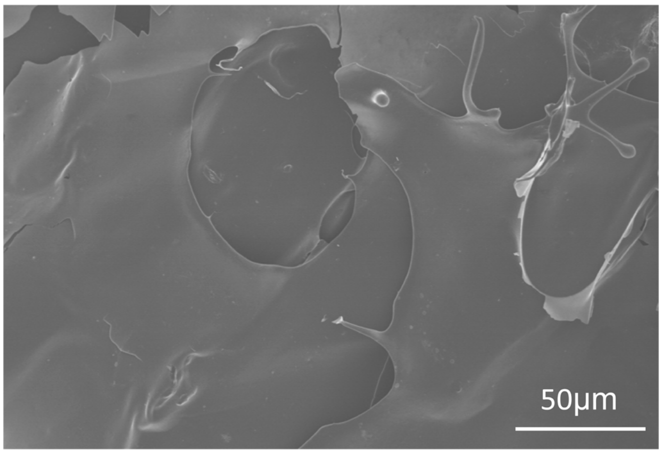

Figure S1. SEM image of CSTB-0.

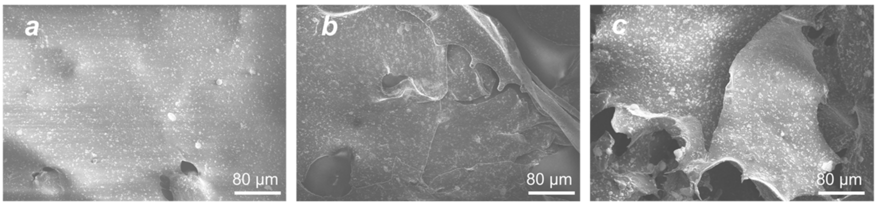

Figure S2. SEM images of the (a) CSTB-0.5, (b) CSTB-1.0 and (c) CSTB-1.5.

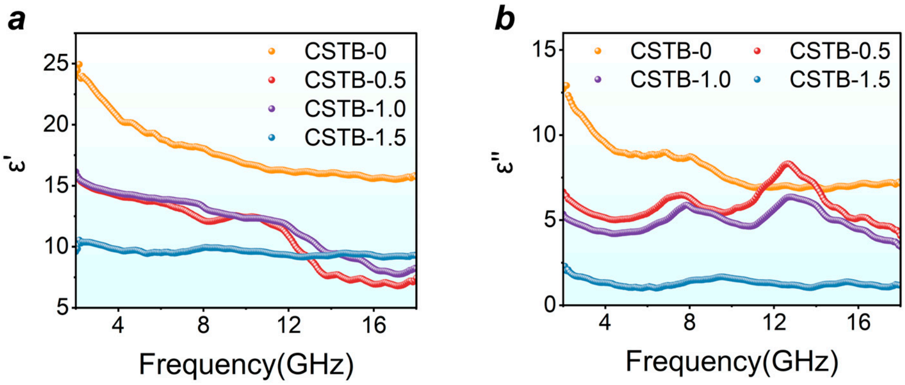

Figure S3. The (a) real and (b) imaginary parts of the complex permittivity of the $BaTiO_3$@C nanocomposites.

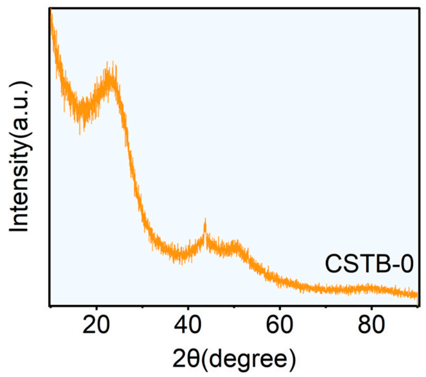

Figure S4. XRD patterns of CSTB-0.

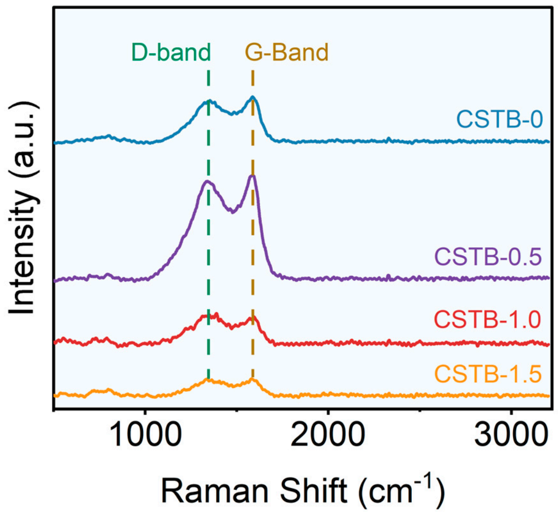

Figure S5. Raman spectroscopy of the $BaTiO_3$@C nanocomposites.

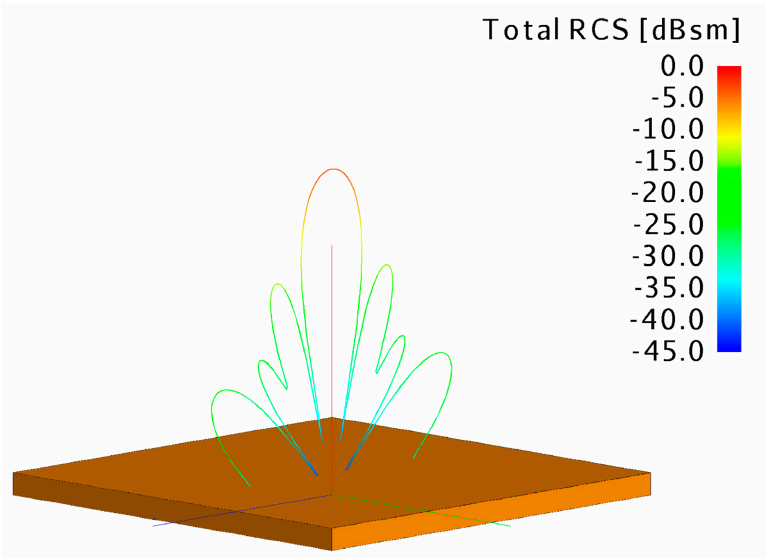

Figure S6. Corresponding RCS simulation results of PEC.

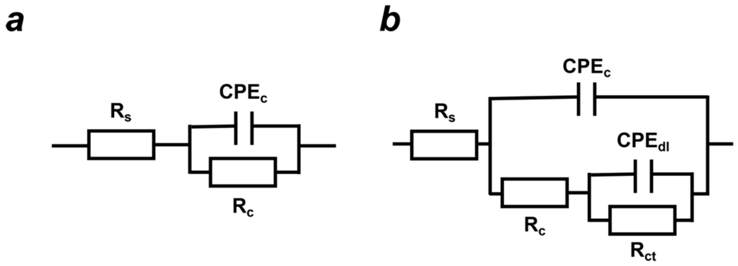

Figure S7. Equivalent circuits used for fitting the EIS spectra: (a) $R_s + (CPE_c \parallel R_c)$ for CSTB-0 and (b) $R_s + [CPE_c \parallel (R_c + (CPE_{dl} \parallel R_{ct}))]$ for CSTB-0.5, CSTB-1.0, and CSTB-1.5.

Table S1. Quantitative comparison of pore structure, average dielectric properties, average attenuation capability, and impedance matching behavior of BaTiO3@C nanocomposites.

	SBET (m^2/g)	Vpore (cm^3/g)	Average pore size (nm)	$\overline{\varepsilon'}$	$\overline{\varepsilon''}$	$\overline{\tan\delta_\varepsilon}$	$\overline{\alpha}$	Impedance matching region (IMR)
CSTB-0	557.5643	0.2240	1.6069	17.7682	8.1177	0.4548	188.2226	8.83%
CSTB-0.5	265.5378	0.1509	2.2740	11.1592	5.9045	0.5690	192.0213	11.17%
CSTB-1.0	127.9394	0.1239	3.8730	11.8968	4.9423	0.4325	155.0398	11.77%
CSTB-1.5	194.1231	0.1548	3.1907	9.5732	1.3133	0.1368	43.2700	1.88%

The dielectric parameters $\overline{\varepsilon'}$, $\overline{\varepsilon''}$, $\overline{\tan\delta_\varepsilon}$, and attenuation constant $\overline{\alpha}$ are average values over the measured frequency range of 2-18 GHz. IMR represents the percentage of calculated frequency-thickness points satisfying IMI < 0.5.

Table S2. Comparison of properties of some typical Chitosan-derived nanocomposites and similar metal/carbon-based absorbers.

Absorbers	RL [dB]	EAB [GHz]	Refs.
MoSe2/RGO/MMT-Chitosan	-72	3.8	[3]
nest-like C/SnO_2	-48.8	5.2	[4]
CoNC-800	-44.3	6.4	[5]
cellulose-chitosan/PANI	-54.76	5.12	[6]
3D carbon aerogels	-41	5.24	[7]
CGCA	-61.67	4.24	[8]
CDC-based aerogels	-69.56	6.56	[9]
CSTB-1.0	-48.07	7.04	This work

Table S3. EIS fitting parameters of CSTB-x samples in 3.5 wt% NaCl solution.

	R_s (Ωcm^2)	CPE_c		R_c ($10^4\Omega$ cm^2)	CPE_{dl}		R_{ct} ($10^4\Omega$ cm^2)	R_c+R_{ct} ($10^4\Omega$ cm^2)
		Y_0 ($10^{-4}\Omega^{-1}$ $cm^{-2}s^n$)	n		Y_0 ($10^{-4}\Omega^{-1}$ $cm^{-2}s^n$)	n		
CSTB-0	2.420	3.478	0.818	4.811	-	-	-	4.811
CSTB-0.5	2.286	2.635	0.776	0.2147	5.154	0.757	6.375	6.590
CSTB-1.0	2.388	3.953	0.835	1.061	6.450	0.944	5.572	6.633
CSTB-1.5	2.327	3.357	0.804	1.698	25.65	1.000	0.6735	2.372

Table S4. Fitted electrochemical parameters obtained from Tafel polarization curves of CSTB-x samples in 3.5 wt% NaCl solution.

	**E_{corr} (V vs. Ag/AgCl)**	**I_{corr} (10-6A·cm^{-2})**	**R_p (Ω·cm^2)**
CSTB-0	-0.0584	20.933	4084
CSTB-0.5	-0.1087	10.63	6657.1
CSTB-1.0	-0.0999	8.9346	7866.1
CSTB-1.5	-0.1483	19.76	5991.9

References

1. C.F. Holder, R.E. Schaak, Tutorial on Powder X-ray Diffraction for Characterizing Nanoscale Materials, ACS Nano 13 (2019) 7359-7365. https://doi.org/10.1021/acsnano.9b05157.
2. A.A. Bunaciu, E.G. Udriştioiu, H.Y. Aboul-Enein, X-Ray Diffraction: Instrumentation and Applications, Crit. Rev. Anal. Chem. 45 (2015) 289-299. https://doi.org/10.1080/10408347.2014.949616.
3. M. Dehghani-Dashtabi, H. Hekmatara, M. Mohebbi, Ultralight microwave absorber with an enhanced absorption performance based on chitosan aerogel, Sci. Rep. 16 (2026) 9475. https://doi.org/10.1038/s41598-026-40116-2.
4. J. Lu, Y. Wang, L. Wang, D. Liu, L. Zhou, C. Wei, X. Zhang, X. Huang, G. Wen, Construction of chitosan-derived porous nest-like C/SnO2 materials for microwave absorption, Int. J. Biol. Macromol. 254 (2024) 127851. https://doi.org/https://doi.org/10.1016/j.ijbiomac.2023.127851.
5. W. Chu, K. Wang, S. Liu, Y. Chen, H. Li, H. Liu, Tailorable effective microwave absorption bandwidth of chitosan-derived carbon-based aerogel under different compression, Mater. Res. Bull. 177 (2024) 112857. https://doi.org/https://doi.org/10.1016/j.materresbull.2024.112857.
6. Z. Zhang, J. Tan, W. Gu, H. Zhao, J. Zheng, B. Zhang, G. Ji, Cellulose-chitosan framework/polyailine hybrid aerogel toward thermal insulation and microwave absorbing application, Chem. Eng. J. 395 (2020) 125190. https://doi.org/https://doi.org/10.1016/j.cej.2020.125190.
7. J. Xu, Three dimensional carbon aerogel for microwave absorption from chitosan, Synth. Met. 295 (2023) 117352. https://doi.org/https://doi.org/10.1016/j.synthmet.2023.117352.
8. L. Zhang, C. Wang, S. Xie, H. Hu, Lightweight and hierarchical carboxyl chitosan-derived carbon aerogel for electromagnetic wave absorber and heat insulation, Compos. Commun. 51 (2024) 102033. https://doi.org/https://doi.org/10.1016/j.coco.2024.102033.
9. R. Shu, X. Ding, L. Xu, K. Tian, Synthesis of chitosan derived carbon-based magnetic composite aerogels with a unique three-dimensional porous network structure as eco-friendly and high-efficiency electromagnetic wave absorbers, Mater. Today Chem. 49 (2025) 103120. https://doi.org/https://doi.org/10.1016/j.mtchem.2025.103120.

Disclaimer/Publisher's Note: The statements, opinions and data contained in all publications are solely those of the individual author(s) and contributor(s) and not of MDPI and/or the editor(s). MDPI and/or the editor(s) disclaim responsibility for any injury to people or property resulting from any ideas, methods, instructions or products referred to in the content.
